# 2-Amino-5-cyano­pyridinium nitrate

**DOI:** 10.1107/S1600536808028031

**Published:** 2008-09-06

**Authors:** Jing Dai

**Affiliations:** a Ordered Matter Science Research Center, College of Chemistry and Chemical Engineering, Southeast University, Nanjing 210096, People’s Republic of China

## Abstract

In the title compound, C_6_H_6_N_3_^+^·NO_3_^−^, the packing is consolidatedby N—H⋯N and N—H⋯O hydrogen bonds.

## Related literature

For the chemisty of amine derivatives, see: Manzur *et al.* (2007 ▶); Ismayilov *et al.* (2007 ▶); Austria *et al.* (2007 ▶); Wen (2008 ▶).
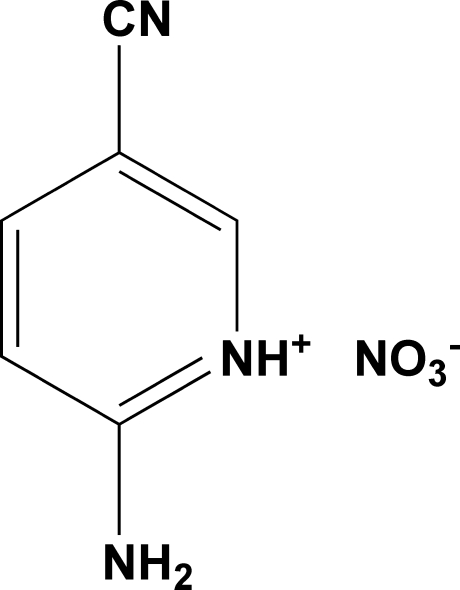


## Experimental

### 

#### Crystal data

C_6_H_6_N_3_^+^·NO_3_^−^
*M* *_r_* = 182.15
Monoclinic, 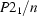

*a* = 4.6475 (9) Å
*b* = 12.713 (3) Å
*c* = 13.417 (3) Å
β = 97.91 (3)°
*V* = 785.1 (3) Å^3^
*Z* = 4
Mo *K*α radiation
μ = 0.13 mm^−1^
*T* = 298 (2) K
0.25 × 0.15 × 0.15 mm

#### Data collection

Rigaku Mercury2 diffractometer
Absorption correction: multi-scan (*CrystalClear*; Rigaku, 2005 ▶) *T*_min_ = 0.975, *T*_max_ = 0.981
8053 measured reflections
1798 independent reflections
1163 reflections with *I* > 2σ(*I*)
*R*_int_ = 0.050

#### Refinement

*R*[*F* ^2^ > 2σ(*F* ^2^)] = 0.053
*wR*(*F* ^2^) = 0.127
*S* = 1.07
1798 reflections
142 parameters
All H-atom parameters refined
Δρ_max_ = 0.17 e Å^−3^
Δρ_min_ = −0.17 e Å^−3^

### 

Data collection: *CrystalClear* (Rigaku, 2005 ▶); cell refinement: *CrystalClear*; data reduction: *CrystalClear*; program(s) used to solve structure: *SHELXS97* (Sheldrick, 2008 ▶); program(s) used to refine structure: *SHELXL97* (Sheldrick, 2008 ▶); molecular graphics: *SHELXTL* (Sheldrick, 2008 ▶); software used to prepare material for publication: *SHELXTL*.

## Supplementary Material

Crystal structure: contains datablocks I, global. DOI: 10.1107/S1600536808028031/wk2092sup1.cif

Structure factors: contains datablocks I. DOI: 10.1107/S1600536808028031/wk2092Isup2.hkl

Additional supplementary materials:  crystallographic information; 3D view; checkCIF report

## Figures and Tables

**Table 1:** Hydrogen-bond geometry (Å, °)

*D*—H⋯*A*	*D*—H	H⋯*A*	*D*⋯*A*	*D*—H⋯*A*
N2—H2*B*⋯O2^i^	0.90 (2)	2.05 (3)	2.941 (3)	169 (2)
N1—H1⋯O1^i^	0.92 (3)	1.82 (3)	2.733 (2)	170 (2)
N2—H2*C*⋯O3	0.83 (3)	2.10 (3)	2.926 (3)	174 (3)

Symmetry code: (i) 
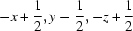
.

**Fig. 1.**
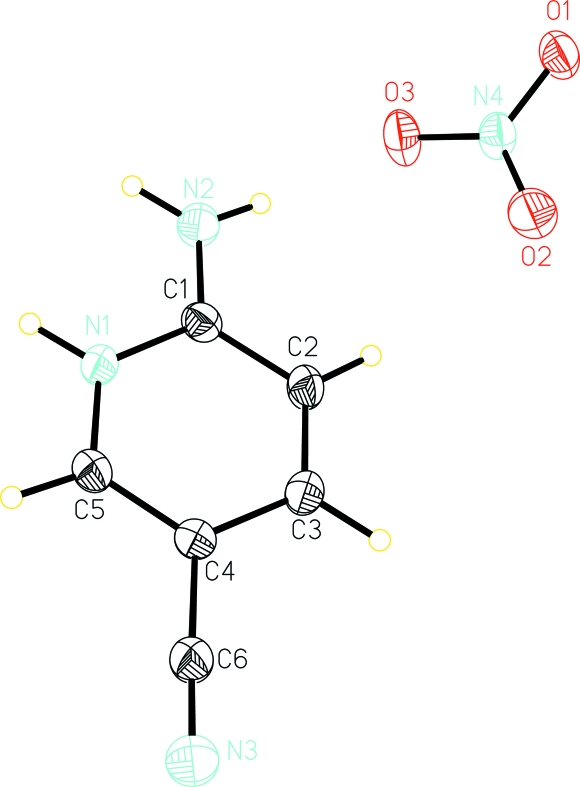
A view of the title compound with the atom numbering scheme. Displacement ellipsoids were drawn at the 30% probability level.

**Fig. 2.**
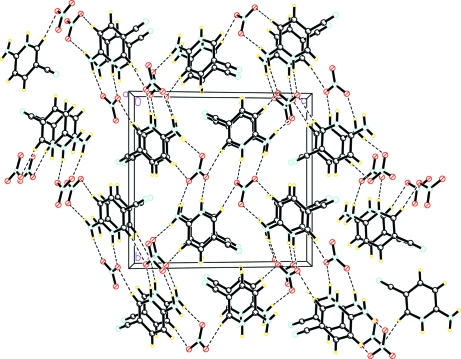
The crystal packing of the title compound viewed along the a axis and all hydrogen atoms not involved in hydrogen bonding (dashed lines) were omitted for clarity.

## supplementary crystallographic information

### Comment

In the past five years, we have focused on the chemistry of amine derivatives because of their multiple coordination modes as ligands to metal ions and for the construction of novel metal–organic frameworks (Manzur *et al.* 2007; Ismayilov *et al.* 2007; Austria *et al.* 2007; Wen 2008). We report here the crystal structure of the title compound.

In the title compound (Fig. 1), the N1 atom of the pyridine ring is protonated. The nitrile group and the pyridinium ring are essentially coplanar. The nitrile group C6≡N3 bond length of 1.133 (3)Å is within the normal range. Crystal cohesion is enforced by N—H···N and N—H···O hydrogen bonds (Table 1, Fig. 2).

### Experimental

6-aminonicotinonitrile (3 mmol) was dissolved in the solution of ethanol (20 ml) and nitric acid (1 ml), and evaporated in the air affording colorless block crystals of this compound suitable for X-ray analysis.

### Refinement

All H atoms were located in difference Fourier maps and refined freely.

### Crystal data

**Table:**

C_6_H_6_N_3_^+^·NO_3_^−^	*F*(000) = 376
*M**_r_* = 182.15	*D*_x_ = 1.541 Mg m^−^^3^
Monoclinic, *P*2_1_/*n*	Mo *K*α radiation, λ = 0.71073 Å
Hall symbol: -P 2yn	Cell parameters from 1796 reflections
*a* = 4.6475 (9) Å	θ = 3.1–27.5°
*b* = 12.713 (3) Å	µ = 0.13 mm^−^^1^
*c* = 13.417 (3) Å	*T* = 298 K
β = 97.91 (3)°	Block, colourless
*V* = 785.1 (3) Å^3^	0.25 × 0.15 × 0.15 mm
*Z* = 4	

### Data collection

**Table:**

Rigaku Mercury2 diffractometer	1798 independent reflections
Radiation source: fine-focus sealed tube	1163 reflections with *I* > 2σ(*I*)
graphite	*R*_int_ = 0.050
Detector resolution: 13.6612 pixels mm^-1^	θ_max_ = 27.5°, θ_min_ = 3.1°
ω scans	*h* = −6→6
Absorption correction: multi-scan (CrystalClear; Rigaku, 2005)	*k* = −16→16
*T*_min_ = 0.975, *T*_max_ = 0.981	*l* = −17→17
8053 measured reflections	

### Refinement

**Table:**

Refinement on *F*^2^	Primary atom site location: structure-invariant direct methods
Least-squares matrix: full	Secondary atom site location: difference Fourier map
*R*[*F*^2^ > 2σ(*F*^2^)] = 0.053	Hydrogen site location: difference Fourier map
*wR*(*F*^2^) = 0.127	All H-atom parameters refined
*S* = 1.07	*w* = 1/[σ^2^(*F*_o_^2^) + (0.0514*P*)^2^ + 0.1542*P*] where *P* = (*F*_o_^2^ + 2*F*_c_^2^)/3
1798 reflections	(Δ/σ)_max_ < 0.001
142 parameters	Δρ_max_ = 0.17 e Å^−^^3^
0 restraints	Δρ_min_ = −0.17 e Å^−^^3^

### Special details

**Table:**

Geometry. All e.s.d.'s (except the e.s.d. in the dihedral angle between two l.s. planes) are estimated using the full covariance matrix. The cell e.s.d.'s are taken into account individually in the estimation of e.s.d.'s in distances, angles and torsion angles; correlations between e.s.d.'s in cell parameters are only used when they are defined by crystal symmetry. An approximate (isotropic) treatment of cell e.s.d.'s is used for estimating e.s.d.'s involving l.s. planes.
Refinement. Refinement of *F*^2^ against ALL reflections. The weighted *R*-factor *wR* and goodness of fit *S* are based on *F*^2^, conventional *R*-factors *R* are based on *F*, with *F* set to zero for negative *F*^2^. The threshold expression of *F*^2^ > σ(*F*^2^) is used only for calculating *R*-factors(gt) *etc*. and is not relevant to the choice of reflections for refinement. *R*-factors based on *F*^2^ are statistically about twice as large as those based on *F*, and *R*- factors based on ALL data will be even larger.

### Fractional atomic coordinates and isotropic or equivalent isotropic displacement parameters (Å^2^)

**Table:**

	*x*	*y*	*z*	*U*_iso_*/*U*_eq_	
C2	0.7591 (5)	0.86151 (17)	0.31844 (17)	0.0428 (5)	
N4	0.0550 (4)	0.94943 (14)	0.12683 (14)	0.0456 (5)	
N2	0.4566 (4)	0.71680 (19)	0.25422 (16)	0.0509 (5)	
O1	−0.1866 (3)	0.98288 (12)	0.08728 (13)	0.0604 (5)	
O2	0.2162 (4)	1.00628 (14)	0.18518 (14)	0.0651 (5)	
O3	0.1266 (4)	0.85819 (13)	0.10891 (13)	0.0610 (5)	
N1	0.8152 (4)	0.69031 (15)	0.38842 (14)	0.0406 (4)	
C3	0.9809 (5)	0.89511 (17)	0.38692 (17)	0.0435 (5)	
C5	1.0356 (4)	0.72284 (18)	0.45750 (17)	0.0402 (5)	
C6	1.3541 (5)	0.86115 (17)	0.53382 (18)	0.0476 (6)	
C1	0.6718 (4)	0.75525 (16)	0.31857 (15)	0.0379 (5)	
C4	1.1235 (4)	0.82498 (16)	0.45893 (16)	0.0388 (5)	
N3	1.5342 (5)	0.89264 (16)	0.59161 (18)	0.0684 (7)	
H5	1.126 (4)	0.6708 (16)	0.5011 (15)	0.036 (5)*	
H2A	0.655 (4)	0.9049 (16)	0.2716 (15)	0.033 (5)*	
H3	1.041 (4)	0.9695 (19)	0.3867 (16)	0.051 (6)*	
H2B	0.401 (5)	0.650 (2)	0.2641 (17)	0.048 (7)*	
H1	0.764 (5)	0.620 (2)	0.389 (2)	0.067 (8)*	
H2C	0.370 (6)	0.761 (3)	0.216 (2)	0.082 (10)*	

### Atomic displacement parameters (Å^2^)

**Table:**

	*U*^11^	*U*^22^	*U*^33^	*U*^12^	*U*^13^	*U*^23^
C2	0.0473 (13)	0.0351 (11)	0.0432 (12)	0.0020 (9)	−0.0043 (11)	0.0098 (10)
N4	0.0556 (12)	0.0365 (10)	0.0425 (10)	0.0046 (9)	−0.0013 (9)	0.0013 (9)
N2	0.0512 (12)	0.0424 (12)	0.0529 (12)	−0.0048 (10)	−0.0146 (10)	0.0011 (10)
O1	0.0532 (10)	0.0445 (9)	0.0766 (12)	0.0102 (8)	−0.0156 (9)	−0.0064 (8)
O2	0.0615 (11)	0.0566 (11)	0.0698 (11)	0.0031 (8)	−0.0179 (9)	−0.0180 (9)
O3	0.0770 (12)	0.0405 (9)	0.0599 (11)	0.0171 (8)	−0.0098 (9)	−0.0044 (8)
N1	0.0405 (10)	0.0306 (10)	0.0478 (11)	−0.0032 (8)	−0.0041 (8)	0.0032 (8)
C3	0.0471 (13)	0.0323 (12)	0.0492 (13)	−0.0021 (10)	−0.0003 (11)	0.0029 (10)
C5	0.0394 (11)	0.0383 (12)	0.0408 (12)	0.0034 (9)	−0.0025 (10)	0.0052 (10)
C6	0.0495 (14)	0.0373 (12)	0.0515 (14)	−0.0002 (10)	−0.0089 (12)	0.0008 (10)
C1	0.0356 (11)	0.0399 (12)	0.0370 (11)	0.0027 (9)	0.0007 (9)	0.0003 (9)
C4	0.0355 (11)	0.0377 (12)	0.0414 (11)	−0.0002 (9)	−0.0014 (9)	0.0020 (10)
N3	0.0697 (14)	0.0501 (13)	0.0755 (16)	−0.0045 (11)	−0.0249 (13)	−0.0036 (11)

### Geometric parameters (Å, °)

**Table:**

C2—C3	1.352 (3)	N1—C5	1.348 (3)
C2—C1	1.411 (3)	N1—C1	1.354 (3)
C2—H2A	0.92 (2)	N1—H1	0.92 (3)
N4—O3	1.239 (2)	C3—C4	1.411 (3)
N4—O2	1.239 (2)	C3—H3	0.99 (2)
N4—O1	1.249 (2)	C5—C4	1.361 (3)
N2—C1	1.322 (3)	C5—H5	0.94 (2)
N2—H2B	0.90 (2)	C6—N3	1.133 (3)
N2—H2C	0.83 (3)	C6—C4	1.440 (3)
			
C3—C2—C1	119.6 (2)	C2—C3—H3	119.4 (13)
C3—C2—H2A	123.7 (12)	C4—C3—H3	120.0 (13)
C1—C2—H2A	116.7 (12)	N1—C5—C4	120.1 (2)
O3—N4—O2	120.89 (19)	N1—C5—H5	116.3 (12)
O3—N4—O1	119.08 (18)	C4—C5—H5	123.6 (12)
O2—N4—O1	120.00 (18)	N3—C6—C4	177.9 (2)
C1—N2—H2B	117.2 (15)	N2—C1—N1	118.9 (2)
C1—N2—H2C	115 (2)	N2—C1—C2	123.1 (2)
H2B—N2—H2C	127 (3)	N1—C1—C2	118.07 (19)
C5—N1—C1	123.0 (2)	C5—C4—C3	118.77 (19)
C5—N1—H1	117.7 (16)	C5—C4—C6	120.53 (19)
C1—N1—H1	119.4 (17)	C3—C4—C6	120.69 (19)
C2—C3—C4	120.5 (2)		
			
C1—C2—C3—C4	−0.6 (3)	C3—C2—C1—N1	0.2 (3)
C1—N1—C5—C4	−0.2 (3)	N1—C5—C4—C3	−0.3 (3)
C5—N1—C1—N2	−179.4 (2)	N1—C5—C4—C6	178.5 (2)
C5—N1—C1—C2	0.2 (3)	C2—C3—C4—C5	0.6 (3)
C3—C2—C1—N2	179.8 (2)	C2—C3—C4—C6	−178.2 (2)

### Hydrogen-bond geometry (Å, °)

**Table:**

*D*—H···*A*	*D*—H	H···*A*	*D*···*A*	*D*—H···*A*
N2—H2B···O2^i^	0.90 (2)	2.05 (3)	2.941 (3)	169 (2)
N1—H1···O1^i^	0.92 (3)	1.82 (3)	2.733 (2)	170 (2)
N1—H1···N4^i^	0.92 (3)	2.62 (3)	3.505 (3)	161 (2)
N2—H2C···O3	0.83 (3)	2.10 (3)	2.926 (3)	174 (3)

Symmetry codes: (i) −*x*+1/2, *y*−1/2, −*z*+1/2.
